# MoOx-Based Colorimetric Sensor for Ultraviolet Visualization

**DOI:** 10.3390/molecules29071486

**Published:** 2024-03-27

**Authors:** Zhaokang Zheng, Zhen Liu, Xingying Li, Aiwu Wang

**Affiliations:** College of Engineering Physics, Shenzhen Technology University, Shenzhen 518118, China; 2210412012@stumail.sztu.edu.cn (Z.Z.); 202200802007@stumail.sztu.edu.cn (Z.L.); 202200703044@stumail.sztu.edu.cn (X.L.)

**Keywords:** ultraviolet sensors, MoOx nanostructures, photochromic compounds, visual analysis, visualization

## Abstract

Due to the depletion of the global ozone layer and the presence of ozone holes, humans are increasingly exposed to threats from solar ultraviolet radiation. Therefore, researching and developing a highly selective, sensitive, simple, and fast ultraviolet sensor is of significant importance for personal protection. In recent years, new nanomaterials have shown good application prospects in the research of ultraviolet sensors. MoOx nanostructures were prepared by a hydrothermal method. The experimental results show that, compared to traditional photochromic compounds, the new MoOx nanostructures exhibit high uniqueness, high selectivity, and excellent stability, and can perform rapid and accurate detection under full-band light. The beam sensor can not only detect through traditional electrical signal output, but also amplify, display, and analyze the beam through visualization and visual analysis, further improving the reliability and practicality of its application.

## 1. Introduction

As is widely known, ultraviolet (UV) radiation is of paramount importance, extensively utilized in various aspects of human daily life, medicine, agriculture, and industrial manufacturing, such as for disinfection, promoting vitamin D synthesis, enhancing plant photosynthesis, and extreme ultraviolet lithography [1,2,3]. Short−term overexposure to solar UV radiation can lead to sunburn and impaired vision, while prolonged exposure increases the likelihood of skin cancer, cataracts, and other diseases [4,5]. However, UV radiation also offers certain benefits and has found applications in the prevention or treatment of rickets and psoriasis [6,7]. Therefore, determining the level of UV radiation is crucial for balancing the beneficial and detrimental effects of sunlight exposure. Hence, the development of a highly selective, sensitive, simple, and fast solar UV radiation sensor holds significant importance for humanity, especially for those engaged in outdoor work or activities.

UV sensors developed in recent years can be broadly categorized into semiconductor photodiode-based sensors and colorimetric sensors [8,9,10] The former has high sensitivity and stability, but their high cost and cumbersome preparation process limit their use in large−scale applications [11,12], in contrast to the latter, which are relatively simple to prepare and allow the visualization of real-time UV irradiation data [13,14]. In recent years, the development of nanomaterials has provided new ideas for the preparation of high-performance and low-cost UV sensors [15,16,17,18,19]. Nano MoOx materials have received much attention in the field of sensors due to their excellent electrical and optical properties [20,21]. Compared to traditional photochromic compounds such as diarylethene derivatives [22], spiropyran derivatives [23], and salicylaldehyde Schiff bases [24], nano MoOx materials exhibit better stability and cyclic performance. However, the current nano MoOx sensors still have some problems, such as insufficient sensitivity and stability and complicated preparation methods [25,26]. In order to solve these problems, this paper proposes a visual UV sensor based on MoOx nanostructures, which is based on the combination of the color change induced by MoOx nanostructures under UV irradiation and machine vision. The core receptor (MoOx nanostructures) of this sensor is simple to prepare and also has excellent sensitivity and stability [27]. In addition, the sensor has a visualization output that allows the user to directly observe the intensity of UV radiation, further enhancing its usefulness and convenience.

## 2. Results and Discussion

### 2.1. UV Absorption Properties of MoOx Nanostructures

**Figure 1:**
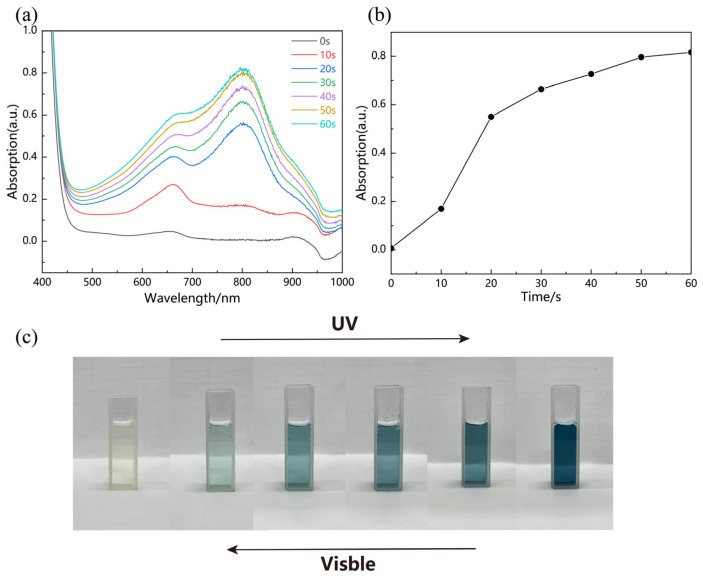
(**a**) UV–visible absorption spectra of MoOx nanostructures exposed to 360 nm UV radiation. (**b**) Change of absorption peak at 800 nm under UV irradiation. (**c**) Photographs of MoOx nanostructures exposed to UV irradiation for 100 s.

For the sample not exposed to ultraviolet light, the spectrum showed that it was light yellow, and no absorption peak was observed in the visible light wavelength range. After receiving UV irradiation, the sample showed a gradual deepening of its color, and its color change is reflected in its UV–visible absorption spectrum (Figure 1a), where the sample changes from light yellow to light blue to dark blue and finally to black (Figure 1c). With increasing exposure time, a broad absorption peak at about 600 nm gradually appeared in the range of less than 1000 nm, while two further peaks were observed at about 650 nm and 800 nm. The appearance of broad absorption peaks indicates a strong localized surface plasmon resonance effect [28,29]. MoOx nanostructures have strong absorption peaks in the UV range.

Ion embedding is the most effective method used to increase the concentration of free carriers in 2D materials to induce plasmon resonance in the visible and near-infrared (NIR) regions [30,31]. The free electron concentration in MoOx can be significantly increased by introducing oxygen vacancies through H+ embedded in the intrinsic intercalation of the material. In addition, the embedded H+ can cause the expansion of the interlayer space, thus increasing the free electron concentration by introducing oxygen vacancies [32,33,34]. NMP is a non-protonic solvent with no hydrogen bonding between the solvent molecules, and also the photo-oxidation of NMP is weak. Therefore, in the NMP/H_2_O system, water is photo-oxidized under UV light and the H+ produced in the process is embedded in MoO_3_. On the other hand, NMP has the highest proton affinity (~920 kJ mol^−1^) [35] and the lowest proton dissociation rate, which results in a higher degree of H+ embedding in NMP compared to other solvents [36].

In summary, the mechanism of ultraviolet (UV) light radiation is summarized as follows [36] (Equation (1) represents the excited state):

MoO_3_ + hv + H_2_O → H_X_MoO_3_-x + O_2_ (1)

Throughout the photo-oxidation process, H_2_O provides a source of hydrogen ions, while NMP acts as a hole−trapping acceptor, which promotes the separation of electrons and holes, thereby accelerating the reaction process. This facilitation leads to an increase in hydrogen ion migration in the system. More explanations about the mechanism of color change of MoOx nanostructures, as well as the morphology and optical testing of MoOx materials, can be found in our previous work [27].

Then, we studied the absorption peak-to-peak value during UV irradiation and made a peak absorptivity versus time plot (Figure 1b) to represent the time gradient change, and found that there was a partially linear region of the peak change and a saturation process of photochromism in this material, which further determines its application in the direction of UV sensors.

### 2.2. Development of UV Colorimetric Sensors

Based on these results, the color of MoOx nanostructured solutions changes from light yellow to dark blue under UV irradiation. Determining the correlation between the color change process and the absorbed ultraviolet radiation is a challenging task due to the involvement of various complex chemical and physical processes. To address this challenge, we introduced visual analysis as a solution. In the field of UV sensors, visual analysis proves to be an effective method as it enables direct observation of the sensor’s response to ultraviolet radiation with the naked eye [37,38]. Analyzing the color change data of the sensor elements allows us to infer important information such as the intensity and wavelength of the ultraviolet radiation. However, due to the typically complex nature of raw data, direct interpretation can be difficult. Therefore, visual analysis plays a crucial role in transforming this data into practical information. Through visual analysis and visualization, UV data can be converted into an easy-to-understand graphical form to help people understand the meaning and characteristics of UV more intuitively [39,40].

Our designed machine vision algorithm was implemented using Pycharm software (version 3.1.0.) running Python code to process the UV sensor data. First, the program read the captured image and extracted the desired portion of the image through the selected region (Supplementary Materials). Then, the image was converted to HSV space, the mask of the target region was obtained by thresholding, and the average of the pixel values within the region was calculated as the value detected by the UV sensor. In addition, the program implemented data saving and visualization. The Excel data were written through the xlwt module, and the time and value were stored in the Excel table, respectively, which was convenient for subsequent data analysis. At the same time, line graphs were drawn through the matplotlib.pyplot module to visualize and display the data, which helps the user to understand the trends of data changes more intuitively. After the design of the algorithm for machine vision [41] was completed, we conducted more tests on the UV photochromic process of MoOx nanostructured solutions.

**Figure 2:**
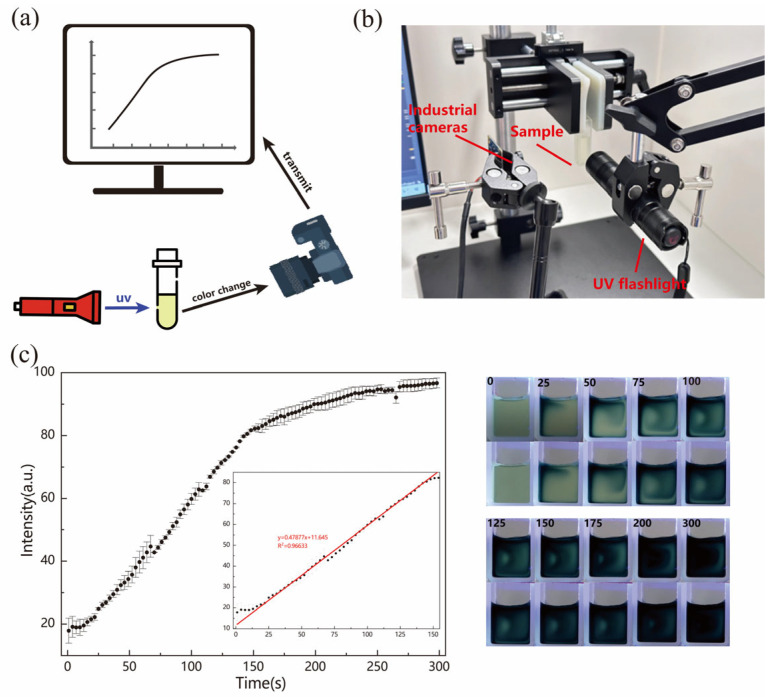
(**a**) Schematic diagram of the test flow. (**b**) Sensor receiver and information acquisition part. (**c**) Function curves obtained by visual analysis and the corresponding photographs. (The numbers in the picture represent the duration of UV exposure for the samples).

When the MoOx nanostructured solution was subjected to UV irradiation, the sample was sampled with an industrial camera (MV-CS060-10GC color (Hikvision, Hangzhou, China)) at a sampling frequency of 30 frames per second, and then analyzed with the designed machine vision algorithm, comprising the whole testing process (Figure 2a); the actual testing process is shown in Figure 2b. The sample was irradiated with a UV flashlight for 300 s, and the results obtained are shown in Figure 2c. The MoOx nanostructured solution had a better linearity before 150 s, and the equation obtained by fitting it is y = 0.4788x + 11.6443. After obtaining the relationship between the amount of absorbed UV radiation and the degree of discoloration, we can judge the degree of discoloration of the sample according to the degree of UV exposure to the radiation. Using the black light radiometer (UVV405) under the test environment of the sample location, the intensity of ultraviolet radiation was found to be 32 mW/cm^2^; after the calculation, the amount of ultraviolet radiation as determined obtained by the sample per second was 48 mJ.

**Figure 3:**
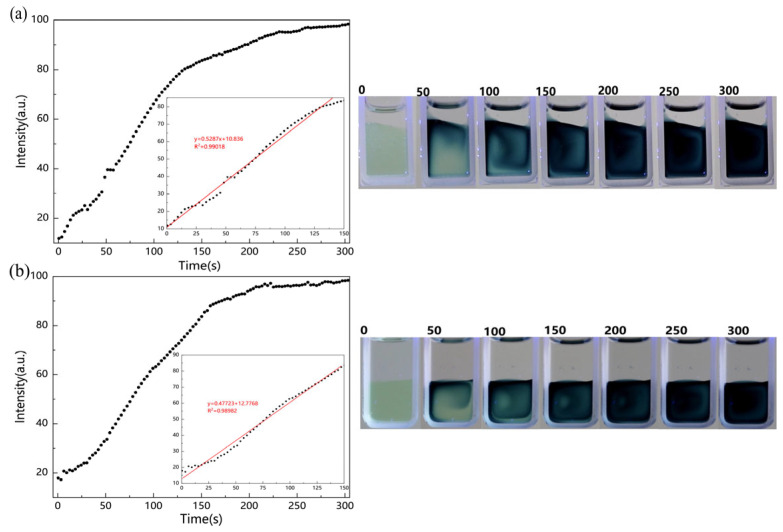
(**a**) Test results of MoOx nanostructured material filled with nitrogen and sealed with silicone oil after boiling. (**b**) Test results of MoOx nanostructured material sealed with silicone oil only. (The numbers in the picture represent the duration of UV exposure for the samples).

We boiled the MoOx nanostructure solution before filling it with nitrogen and sealing it with silicone oil. This test environment reduces the dissolved oxygen in the sample solution and isolates it from the external environment. The results in this test environment are shown in Figure 3a, and again we fitted the equation y = 0.5287x + 10.836 to the interval 0–150 s. This indicates that the UV absorption of the sample is accelerated by removing the dissolved oxygen from the sample, which is also consistent with the principle of discoloration discussed earlier. The results of sealing the MoOx nanostructures with silicone oil only and then testing them are shown in Figure 3b. Fitting the interval segment of 0–150 s yields the equation y = 0.47723x + 12.7768, which is similar to the results of the MoOx nanostructure solution tested directly without treatment. This indicates that sealing with silicone oil has little effect on the UV absorption of the sample.

**Figure 4:**
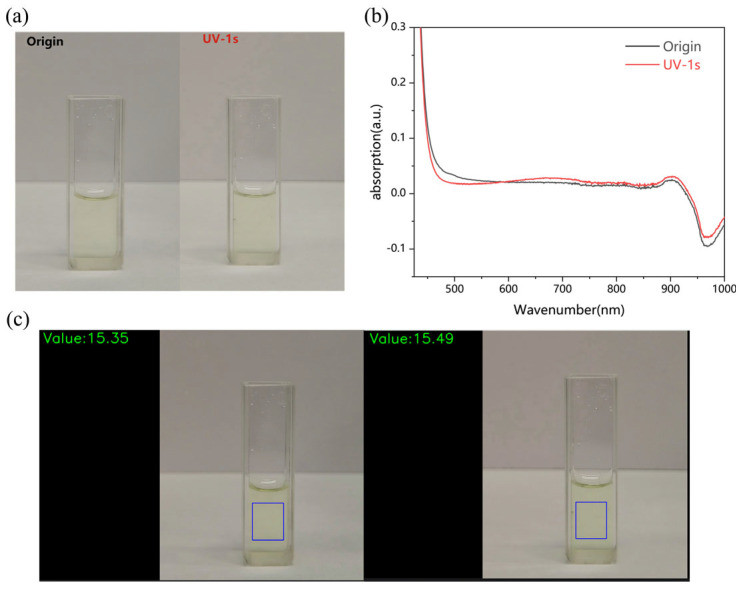
(**a**) Unirradiated MoOx nanostructured solution versus MoOx nanostructured solution irradiated by UV flashlight for 1 s. (**b**) UV−visible absorption spectra before and after UV irradiation. (**c**) Test chart of visual analysis program before and after UV irradiation. (“The blue box” represents the recognition range of the machine vision identification program).

A UV flashlight was used for 1 s to simulate the response of the MoOx nanostructured solution to a small amount of UV light, and the intensity of UV irradiation was 5.1 mW/cm^2^ when the sample was 10 cm away from the UV flashlight as measured by a black light radiometer. The photographs of the sample before and after irradiation by the UV flashlight are shown in Figure 4a, which shows that no difference can be seen when looking at these two photographs, and there is only a slight change in UV–visible absorption spectra (Figure 4b) before and after irradiation. The absorption spectra (Figure 4b) also show only slight changes. However, when these two photos were analyzed by the visual analysis program, two different values were obtained (Figure 4c), which means that our visual recognition program can distinguish the difference between the two photos. On a cloudy day, the measured UV radiation intensity of sunlight is 60 μW/cm^2^; after 6 s of irradiation under this low–ultraviolet–radiationintensity condition, our visual analysis program could identify the color change in MoOx nanostructures. This demonstrates the high sensitivity of the MoOx colorimetric sensor we designed.

## 3. Materials and Methods

### 3.1. Materials

MoO_3_ powder (99%) and N–methyl–2–pyrrolidone (NMP, 98%) were purchased from Aladdin (Shanghai, China). Deionized water (18 MΩ) was used for all experimental processes, and all reagents were used as the original.

### 3.2. Preparation of MoOx Nanostructures

MoO_3_ powder (0.03 g) was added to 10 mL of solvent containing deionized water and NMP (1:1 by volume). The mixture was then transferred to a magnetically heated stirring kettle and heated at 120 °C for 3 h. Subsequently, the sample was placed in a centrifugation chamber at 10,000 rpm for 10 min and supernatant was obtained from the sample.

### 3.3. Photochromic Experiments with MoOx Nanostructures

The MoOx nanostructure solution was irradiated with artificial UV light (Perfectlight, MICROSOLAR300, Beijing, China) for each irradiation time of 10 s. Then, we measured the visible absorption spectrum (Vis, MA, Evolution 220, (Waltham, MA, USA)).

## 4. Conclusions

In summary, for the first time, a visual colorimetric UV sensor based on MoOx nanostructures has been developed. We have investigated the application of MoOx materials in UV sensors through preparation and testing. This includes the preparation of the MoOx material and a discussion of the principle of photochromism. Various experiments were also conducted to test the properties of the MoOx material, including photosensitive response tests. The experimental results show that the MoOx material has excellent properties and is suitable for use in UV sensors. We also designed a UV sensor based on MoOx material using a visual analysis algorithm, and specifically analyzed and evaluated its performance. The sensor’s recognition accuracy is much higher than that of the human eye, it still responds to UV radiation with an intensity of 60 μW/cm^2^, and it is also capable of accomplishing real-time detection of UV light for a long time period and determining the amount of UV radiation absorbed during that time period. Considering the importance of monitoring UV radiation, the results obtained by our MoOx colorimetric sensor are particularly important and may be used on a large scale in areas such as agricultural production.

## Data Availability

The data that support the findings of this study are available from the corresponding author upon reasonable request.

## Supplementary Materials

The following supporting information can be downloaded at: https://www.mdpi.com/article/10.3390/molecules29071486/s1.
