# Supplementary material for: MoOx-Based Colorimetric Sensor for Ultraviolet Visualization
Source: Molecules. 2024 Mar 27;29(7):1486. doi: 10.3390/molecules29071486 (PMC11013073; doi:10.3390/molecules29071486)
Supplement: Supplementary file 1 [file molecules-29-01486-s001.zip › molecules-2752264-supplementary.pdf]

The following is a detailed introduction to the program：

1. Import the required Python modules and libraries

```
import cv2
import xlwt
import time
import numpy as np
import matplotlib.pyplot as plt
```

This imports the cv2 (OpenCV) module for image processing, the xlwt module for saving data to Excel spreadsheets, the time module for getting the current time, the numpy library for mathematical calculations, and the matplotlib.pyplot module for data visualization.

2. Set the graphical interaction mode for data visualization, initialize data lists, create video capture objects to read video frames, select areas of interest, and process images in the area

```
plt.ion()
plt.figure(1)

t_list = []
result_list = []

checkbox = (272, 119, 349, 286)

cap = cv2.VideoCapture(0)
```

This involves activating the interactive mode for real-time data visualization and creating a graphical window to display real-time data trends. The area of interest selected is processed for image analysis. 'cap = cv2.VideoCapture(0)' is used to open the camera and create a video capture object for reading video frames.

3. Convert image color space to HSV and perform threshold processing

```
hsv = cv2.cvtColor(ROI, cv2.COLOR_BGR2HSV)
lower_blue = np.array([100, 50, 50])
upper_blue = np.array([130, 255, 255])
mask = cv2.inRange(hsv, lower_blue, upper_blue)
```

Here, the cv2.cvtColor() function is used to convert the ROI from BGR color space to HSV color space. Then, by setting the lower_blue and upper_blue threshold ranges, the cv2.inRange() function is used to convert the HSV image into a binary mask (mask), where the pixel value of the target area is 255, and the non-target area is 0."

4. Calculate the Average Pixel Value in the Target Area

```
mean_value = cv2.mean(hsv, mask=mask)
```

Here, the cv2.mean() function is used to calculate the average value of the pixels that are 255 (i.e., the target area) in the mask. This average value represents the numerical readings detected by the ultraviolet sensor.

5. Save Data to an Excel Spreadsheet

```
workbook = xlwt.Workbook(encoding='utf-8')
worksheet = workbook.add_sheet('UV_data')
worksheet.write(0, 0, 'Time')
worksheet.write(0, 1, 'UV_value')
row = 1
while True:
    now = time.strftime('%Y-%m-%d %H:%M:%S')
    worksheet.write(row, 0, now)
    worksheet.write(row, 1, mean_value[2])
    workbook.save('UV_data.xls')
    row += 1
    time.sleep(60)
```

Here, the xlwt library is used to create a new Excel spreadsheet. In the first row, two column titles, 'Time' and 'UV_value', are written. Then, through a while loop, data is read every 60 seconds. The current time and mean_value[2] (i.e., the numerical value detected by the ultraviolet sensor) are written into the Excel spreadsheet. Finally, the Excel spreadsheet is saved to a file named 'UV_data.xls'.

6. Plot Visualization of Data on a Line Graph

```
        plt.clf()
        plt.plot(t_list, result_list)
        plt.xlabel('time')
        plt.ylabel('value')
        plt.draw()
```

A trend graph is plotted to show the variation of ultraviolet detection values over time, and the data image is displayed in real-time.
